# Measurement properties of the German version of the birth satisfaction scale-revised (BSS-R) in women with pre-existing medical conditions and high-risk pregnancy

**DOI:** 10.1186/s12884-025-07571-7

**Published:** 2025-04-24

**Authors:** Elena Jost, Waltraut M. Merz, Philipp Kosian, Claudia Hartmann, Matthias Schmid, Caroline J. Hollins Martin, Colin R. Martin

**Affiliations:** 1 Department of Obstetrics and Prenatal Medicine, University Hospital Bonn, Venusberg-Campus 1, 53127 Bonn, Germany; 2 Department of Psychosomatic Medicine, Center of Internal Medicine and Dermatology, Charité - Universitätsmedizin Berlin, Berlin, Germany; 3 Department of Medical Biometry, Informatics, and Epidemiology, University Hospital Bonn, Bonn, Germany; 4 School of Health and Social Care, Edinburgh Napier University, Scotland, UK; 5 Institute for Health and Wellbeing, University of Suffolk, Ipswich, UK

**Keywords:** Birth experience, Birth satisfaction, Patient-reported outcome, Obstetric medicine, Pre-existing condition, High-risk pregnancy, Psychometrics, Quality of intrapartum care

## Abstract

**Background:**

The Birth Satisfaction Scale-Revised (BSS-R) is a validated questionnaire for assessment of childbirth experience which has been translated into many languages. It is the instrument of choice in the International Consortium for Health Outcomes Measurement (ICHOM) standard set for ‘Pregnancy and Childbirth’. Translation of the key outcome measures from English into German language was previously performed, but its validation is pending.

**Aim and Objectives:**

To analyze the key psychometric properties of the German version of the BSS-R (Ger-BSS-R), and to evaluate its application in women with chronic conditions.

**Methods:**

248 women with pre-existing medical conditions were provided with the Ger-BSS-R during hospital inpatient stay for childbirth. The 10-item measurement contains three sub-scales for assessing quality of care provision (QC), women’s personal attributes (WA), and stress experienced during labor (SE).

**Results:**

Complete data was available in *N* = 224 cases. After removal of four multivariate outliers, *N* = 220 were available for psychometric evaluation. The cesarean section rate was 50.5%, prematurity occurred in 14.5% of deliveries and induction of labor was performed in 49.7% of cases with planned vaginal delivery. Mean total BSS-R score was 25.7 (SD 5.94). In the confirmatory factor analysis, the tri-dimensional measurement model was found to offer a good fit to Ger-BSS-R data. For internal consistency, the total, SE and QC sub-scale Cronbach’s alphas were significantly lower than those of the founder version.

**Conclusions:**

The Ger-BSS-R is a robust instrument for assessing birth satisfaction. This is the first study to apply the BSS-R in women with pre-existing medical conditions. Compared to the founder UK version, differences in total BSS-R scores and sub-scales for experience of stress and quality of care are present, requiring further investigations.

**Supplementary Information:**

The online version contains supplementary material available at 10.1186/s12884-025-07571-7.

## Introduction

Women’s experience of childbirth is known to influence long-term health as well as family relationship. Traumatic childbirth is an established risk factor for the development of postpartum mental disorders and impaired infant bonding and partnership [1, 2]. Understanding women’s perceptions of childbirth and identifying factors influencing birth satisfaction are indispensable to improve the quality of health care provision. The World Health Organization (WHO) guidelines on antenatal and intrapartum care underline the importance of a positive birth experience to promote maternal long-term mental health and improve woman-centered health care [3, 4]. Therefore, the standardized assessment of birth satisfaction has become a key instrument for the evaluation of maternity care [5].

According to the WHO, a woman’s personal and sociocultural beliefs and expectations; the birth of a healthy baby; safe environment; emotional support; and competent clinical staff are components of a positive childbirth experience [4]. Various factors of intrapartum care have been identified to influence birth satisfaction such as mode of delivery, intrapartum interventions, pain management, delivery-associated complications, birth place, involvement in decision making, and communication with health care professionals [5–11]. Furthermore, sociodemographic variables, parity, quality of antenatal care, and attendance of antenatal classes may influence a woman’s birth experience [5, 8, 12, 13]. Waldenström et al. (2004) worked out four risk categories for a negative childbirth experience, namely complication-associated factors (e.g., emergency cesarean section, labor augmentation, neonatal intensive care unit admission), sociocultural background-associated factors (e.g., unwanted pregnancy, lack of support), woman’s perception-associated factors (e.g., pain or loss of control) and caregiver-associated factors (e.g., communication, support, analgesia) [14]. Conversely, a medically classified ‘uncomplicated vaginal birth’ may result in a negative birth experience, while women suffering severe intrapartum complications may report a high birth satisfaction [15, 16].

The heterogeneity of influencing factors illustrates that birth satisfaction is a complex, highly subjective concept; no consensus on the definition has yet been reached. As a consequence, the many tools attempting to assess birth satisfaction vary greatly and show lack of uniformity [15].

The Birth Satisfaction Scale-Revised (BSS-R) is a widely utilized instrument to measure birth satisfaction. Its 10-item questionnaire was established by Hollins Martin and Martin in 2014 as a robust, reliable, and valid multidimensional instrument to standardize the assessment of women’s perception of birth experience [17]. The International Consortium for Health Outcomes Measurement (ICHOM) has adopted the BSS-R as part of the standard set ‘Pregnancy and Childbirth’ for the standardized patient-reported outcome (PRO) measures [18].

The BSS-R was drafted in British English. To date, the BSS-R is available in 22 languages, and can therefore be applied to women in over 65 countries (Table S1, supplementary material).

The German translation of the BSS-R has previously been performed and published by Hartmann et al. in 2022 as part of the complete ‘Pregnancy and Childbirth’ ICHOM standard set [19], but has not yet been validated.

The impact of a chronic health condition on birth satisfaction is scarcely investigated, although it is well known that women with pre-existing conditions face higher rates of maternal and perinatal complications [20–22]. Hochman et al. (2023) analyzed risk factors for a negative birth experience. Their cohort (*n* = 1495) included 20 women with a pre-existing condition (type 1 and type 2 diabetes mellitus *n* = 9, and chronic hypertension *n* = 11). No difference in birth experience was found [8]. A systematic literature review by McKelvin et al. (2021) revealed contradictory findings when investigating the impact of mental illness on birth experience [23]. Nonetheless, most studies analyzing risk factors for negative birth experience exclude women with chronic medical conditions from their study cohort [5] although the number of pregnancies in women with pre-existing conditions is rising steadily [24].

This study aims at providing the validation of the German version of the BSS-R, which we phrased Ger-BSS-R in the following. It is also the first study to provide a validation in a cohort of women with pre-existing conditions who therefore were classified as at-risk pregnancies.

## Methods

### Study design

The study is part of a prospective, longitudinal, single-center cohort study on the requirements of care for women with pre-existing conditions (‘ForMaT’). Aim of this study is to integrally assess maternity care in three dimensions, namely maternal and perinatal medical outcome, health-economic data, and women’s perception of pregnancy and becoming a mother in the presence of a pre-existing condition. The complete study protocol was published in 2023 [25].

At the study center, a university hospital regional perinatal center, women with pre-existing conditions are cared for in the framework of a risk-adapted, interdisciplinary setting. From November 2022 until March 2024 women were recruited during their initial presentation, either in the specialized outpatient department for Obstetric Medicine or during inpatient stay.

The ForMaT-Trial was registered in the German Clinical Trials Register (DRKS00030061).

### Instrument and translational process

The BSS-R is a 10-item measurement based on a 5-point Likert scale, scored from 0 to 4. A total score of 40 represents the highest birth satisfaction. This instrument contains three sub-scales for assessing “ quality of care provision (QC), women’s personal attributes (WA), and stress experienced during labour (SE)” [17]. These domains consist of 4, 2, and 4 items respectively and may be calculated individually in addition to the total score calculation.

The previous translation process of the founder BSS-R (British English) into German was performed and published by Hartmann et al. in 2022 [19]. Here, the BSS-R was translated together with other instruments as part of the ICHOM standard set ‘Pregnancy and childbirth’ using the ‘Functional Assessment of Chronic Illness Therapy’ translation method [18, 26]. Cognitive debriefing was performed on 15 postpartum women [19].

### Participants

A detailed description of the characteristics of the study population has been published [27]. Briefly, only women with severe conditions such as a chronic disease (e.g., multiple sclerosis) or history of a health incident (e.g., history of pulmonary embolism) with potential impact on pregnancy, childbirth or women’s perception of pregnancy or childbirth were eligible for inclusion. A list of these conditions can be found in the supplementary material of the study protocol [25]. Participants were required to complete the questionnaire in German language. Women with higher-order multiple pregnancies or women not understanding the German language were not enrolled in the study.

### Data collection

The Ger-BSS-R was either completed tablet-based online with the assistance of a study team member or via paper-and-pencil interview within 72 h after childbirth but before discharge from inpatient stay. Data management and monitoring was executed by the Clinical Support Study Center of the University Hospital Bonn, Germany, and BSS-R data was stored using a specifically build REDCap© database (Version 12.0.27, Nashville, Tennessee, United States) [28, 29].

### Data analysis

Initial screening of the dataset (*n* = 248) for missing Ger-BSS-R data and multivariate outliers was undertaken. Twenty-four cases were removed due to partial missing Ger-BSS-R data. Four multivariate outliers were identified by reference to Mahalanobis distances [30] and removed. The data was analyzed using R (R Core Team, Vienna, Austria) [31].

### Confirmatory factor analysis

Confirmatory Factor Analysis (CFA) was used to evaluate the fit of the established three-factor measurement model comprising correlated SE, WA and QC factors. A bifactor model was also evaluated specified by a global domain of birth experience and three (uncorrelated) domains of SE, WA and QC. A two-factor model [32], combining SE and WA items into a single factor correlated with the QC was also evaluated since a high correlation between SE and WA factors has been observed in a number of studies (e.g. Martin et al. 2018 [33]). The criteria for acceptable model fit were a Comparative Fit Index (CFI) [34] > 0.90, the root mean squared error or approximation (RMSEA) [35] < 0.08 and the square root mean residual (SRMR) [36] < 0.06.

### Divergent validity

Divergent validity was evaluated by correlation of Ger-BSS-R sub-scale scores and the total scale score with participant age [37] and gestation duration [38]. It is predicted that correlations (Pearson’s r) between Ger-BSS-R scores (total and sub-scale) and participant age and gestation duration would be very low (< 0.20) [39].

### Convergent validity

Convergent validity was assessed by comparing Pearson’s r correlations between Ger-BSS-R sub-scale and total scale scores with those reported in the original UK-BSS-R development study [17]. Inferential comparisons were made using the method of Diedenhofen and Musch (2015) [40]. No statistically significant differences between the correlation comparisons were predicted.

### Internal consistency

Cronbach’s alpha [41] was used to assess internal consistency of the Ger-BSS-R sub-scales and total scale, with values of 0.70 or higher considered acceptable [41]. The two items WA sub-scale was assessed for internal consistency using the inter-item correlation (Pearson’s r), with a range of 0.15–0.50 considered acceptable [42]. Calculated alphas were then compared directly with those reported in the original UK-BSS-R development study [17] using the method of Diedenhofen and Musch (2016) [43]. Consistent with contemporary BSS-R translation and validation studies (e.g., Ratislavová et al. 2022 [44]), McDonalds Omega (ω), Omega hierarchical (ωh), and Omega total (ωt) were also calculated to determine internal scale reliability [45, 46].

### Known-groups discriminant validity

Comparisons of Ger-BSSR sub-scale and total scores were used to evaluate Known-Groups Discriminant Validity (KGDV), which is an approach consistent with many BSS-R translation and validation studies (e.g., Grundström et al. 2023 [36]). One-way between-subjects analysis of variance (ANOVA) was used to evaluate group differences in Ger-BSS-R scores. Further, parity was also used to assess KGDV, which is an approach consistent with recent BSS-R translation and validation studies (e.g., Abrán et al. 2024 [37]). The between-subject t-test was undertaken to determine group differences.

## Results

The final dataset for analysis comprised *n* = 220 participants. Table 1 summarizes patient characteristics. Of note, the cesarean section (CS) rate was 50.5%, prematurity occurred in 14.5% of deliveries and induction of labor was performed in 49.7% of cases with planned vaginal delivery.

**Table 1:** Overview of patient characteristics. If not otherwise indicated for characteristics with incomplete data, frequencies and percentages are calculated with *n* = 220. NVD = Normal vaginal delivery, CS = cesarean section

Characteristics		Frequency [*n*]	Percent [%]
Age at delivery [years]	18–34	140	63.6
	≥35	80	36.4
BMI at delivery [kg/cm^2^] (*n* = 155)	< 25	21	13.5
	25-29.9	54	34.8
	30-34.9	44	28.4
	≥35	36	23.2
Nationality (*n* = 215)	German	195	83.3
	Other	20	7.4
Employment (*n* = 214)	yes	168	78.5
	no	46	21.5
Level of education (*n* = 214)	Lower secondary education	56	26.2
	High school degree	59	27.6
	University degree	96	44.9
	No secondary education	3	1.4
Linving in partnership (*n* = 215)	yes	207	96.3
	no	8	3.7
Parity	Primiparous	112	50.9
	Multiparous	108	49.1
History of CS	yes	53	24.1
Gestational age at delivery [weeks]	< 34	6	2.7
	34–36	26	11.8
	≥37	188	85.5
Mode of delivery	NVD	93	42.3
	Instrumental delivery	16	7.3
	CS total	111	50.5
	-emergency CS total	40	18.2
	-emergency repeat CS	10	4.6
	-elective CS total	70	31.8
	-elective repeat CS	34	15.5
Onset of labor (if NVD was attempted, *n* = 145)	Spontaneous	73	50.3
	Induced	72	49.7
Type of anesthesia/analgesia (*n* = 175)	Regional	151	86.3
	General	9	5.1
	Nitrous oxide	15	8.6
Blood loss [ml] (*n* = 217)	< 500	105	48.4
	500–999	84	38.7
	> 1000	28	12.9

### Distributional characteristics

The means, SD, range, skew and kurtosis characteristics of the Ger-BSS-R (items, sub-scales and total score) are shown in Table 2. There was no evidence of excessive skew or kurtosis, though items 5, 6 and 10 had a reduced response range.

**Table 2:** Mean, standard deviation and distributional characteristics of individual Ger-BSS-R items, sub-scale totals and the total Ger-BSS-R score. se = standard error of the mean. *Domain of the Ger-BSS-R. SE = Stress experienced during childbirth, WA = Women’s attributes, QC = Quality of Care

Item	Item content	Domain*	Mean	SD	Min	Max	Skew	Kurtosis	se
1	I came through childbirth virtually unscathed	SE	1.98	1.34	0	4	0.13	-1.26	0.09
2	I thought my labour was excessively long	SE	2.03	1.36	0	4	-0.07	-1.31	0.09
3	The delivery room staff encouraged me to make decisions about how I wanted my birth to progress	QC	2.92	1.09	0	4	-0.81	-0.05	0.07
4	I felt very anxious during my labour and birth	WA	1.53	1.30	0	4	0.57	-0.90	0.09
5	I felt well supported by staff during my labour and birth	QC	3.71	0.59	1	4	-2.31	5.63	0.04
6	The staff communicated well with me during labour	QC	3.72	0.61	1	4	-2.46	6.28	0.04
7	I found giving birth a distressing experience	SE	2.35	1.25	0	4	-0.29	-0.92	0.08
8	I felt out of control during my birth experience	WA	2.12	1.30	0	4	0.00	-1.21	0.09
9	I was not distressed at all during labour	SE	1.52	1.23	0	4	0.53	-0.69	0.08
10	The delivery room was clean and hygienic	QC	3.82	0.44	2	4	-2.39	5.15	0.03
SE	Sub-scale total		7.88	3.47	0	16	0.17	-0.54	0.23
WA	Sub-scale total		3.65	2.21	0	8	0.23	-0.76	0.15
QC	Sub-scale total		14.17	1.93	6	16	-1.37	2.41	0.13
Total	Total score		25.70	5.94	12	40	0.20	-0.46	0.40

### Confirmatory factor analysis

CFA model evaluations are summarized in Table 3. The tri-dimensional measurement model of the BSS-R [17] was found to offer a good fit to Ger-BSS-R data, which is a similar observation for the two-factor model. The chi-square differences test (∆χ^2^ = 4.21, df = 2, *p* = 0.12) revealed no statistically significant difference in model fit between two-factor and three-factor models. The bifactor model also offered a good fit to data. As anticipated, the single-factor model offered a poor fit to data.

**Table 3:** Confirmatory factor analysis and model fit of the Ger-BSS-R. CFI: comparative fit index; RMSEA: root mean squared error or approximation; SRMR: square root mean residual

Model	χ^2^ (df)	*p*	RMSEA	SRMR	CFI
Single factor	254.74	< 0.001	0.169	0.115	0.584
Three-factor	64.63	0.001	0.068	0.059	0.938
Two-factor	68.84	< 0.001	0.068	0.062	0.934
Bifactor	60.13	< 0.001	0.077	0.059	0.935

### Divergent validity

No statistically significant correlations were observed between participant age and SE, WA, QC sub-scales and the total Ger-BSS-R score, *r* = 0.01, *p* = 0.95, *r* = 0.11, *p* = 0.12, *r* = 0.12, *p* = 0.08 and *r* = 0.01, *p* = 0.96 respectively. Examination of the relationship between Ger-BSS-R sub-scale and total scores and gestational age in weeks (mean = 38.07, SD = 2.18) revealed correlations between gestational age in weeks and SE, WA, QC sub-scales and the total Ger-BSS-R score, *r* = 0.05, *p* = 0.48, *r* = 0.16, *p* = 0.02, *r* = 0.11, *p* = 0.12 and *r* = 0.07, *p* = 0.32 respectively to be below the correlational criterion of 0.20.

### Convergent validity

Pearson’s r correlation coefficients between Ger-BSS-R sub-scales and the total Ger-BSS-R score are shown in Table 4. The original UK-BSS-R development study correlations are also shown, and using the correlation comparison method of Diedenhofen and Musch (2015) [39], with the sole exception of correlations between WA and QC sub-scales (*p* = 0.04), no statistically significant differences were observed between the correlation dyads of the current study and those reported in the original instrument development study.

**Table 4:** Correlations of Ger-BSS-R sub-scales and total score and comparison with original UK-BSS-R validation study (Hollins Martin & Martin, 2014)

Scale combination	Current study *r*	UK study *r*	Z	95% CI	*p*
Stress-Attributes	0.63	0.57	0.99	(-0.06–0.18)	0.32
Stress-Quality	0.25	0.26	0.11	(-0.18–0.16)	0.91
Attributes-Quality	0.17	0.35	2.04	(-0.35– -0.01)	0.04
Total score-Stress	0.90	0.86	1.88	(-0.01–0.08)	0.06
Total score-Attributes	0.798*	0.80	0.06	(-0.07–0.07)	0.95
Totals score-Quality	0.54	0.63	1.44	(-0.21–0.03)	0.18

*Correlation at three decimal points to allow formal calculation with UK value

### Internal consistency

Cronbach’s alpha for the total Ger-BSS-R scale were > 0.70. However, Ger-BSS-R sub-scale alphas were all < 0.70 (Table 5). The Ger-BSS-R total, SE and QC sub-scale alphas were significantly lower than those reported by Hollins Martin and Martin (2014) [17]. The inter-item correlation between the two WA items was *r* = 0.47, *p* < 0.05. McDonalds Omega (ω), Omega hierarchical (ωh) and Omega total (ωt), respectively were, 0.76 (95% confidence interval 0.53–0.80), 0.40 and 0.81.

**Table 5:** Cronbach’s alpha of Ger-BSS-R sub-scales and total score and comparison with the original UK BSS-R validation study (Hollins Martin & Martin, 2014). Degrees of freedom = 1

Subscale	Current study	UK study	χ^2^	*p*
Stress	0.59	0.71	3.98	0.05
Attributes	0.62	0.64	0.05	0.82
Quality	0.54	0.74	10.73	0.001
Total score	0.72	0.79	3.76	0.05

### Known-group discriminant validity

Comparisons of Ger-BSS-R sub-scale and total scores as a function of delivery type are shown in Table 6. All ANOVA’s were statistically significant. SE sub-scales were significantly higher in the NVD and elective CS groups compared to emergency CS. QC scores were significantly higher in the NVD group compared to elective CS. The total Ger-BSS-R score was significantly higher in the NVD group compared to the emergency CS group. Though the overall ANOVA finding was statistically significant for the WA sub-scale, none of the multiple comparisons with Bonferroni correction were statistically significant.

**Table 6:** Comparison of Ger-BSS-R total and sub-scale scores differentiated by mode of birth. Due to the small sample size of the vacuum delivery group, this category was excluded from Inferential statistical testing. Standard deviations are in parentheses, degrees of freedom = 2, 200. Subscales: SE = stress experienced during childbirth, WA = women’s attributes, QC = quality of care

BSS-*R* Scale	NVD (*n* = 93) M (SD)	Vacuum Delivery (*n* = 16) M (SD)	Elective CS (*n* = 70) M (SD)	Emergency CS (*n* = 40) M (SD)	F	*p*	ω^2^	95%CI	Effect size
SE	8.16 (3.38)^a^	5.81 (2.24)	8.74 (1.75)^b^	6.50 (6.12)^a, b^	5.59	0.004	0.04	0.00–0.11	Small
WA	4.09 (2.01)	3.44 (2.39)	3.34 (2.31)	3.25 (5.16)	3.20	0.04	0.02	0.00–0.07	Small
QC	14.59 (1.65)^a^	14.13 (2.14)	13.69 (1.74)^a^	14.02 (5.38)	4.83	0.009	0.04	0.00–0.10	Small
Total score	26.84 (5.75)^a^	23.38 (2.13)	25.77 (2.30)	23.78 (6.20)^a^	3.79	0.02	0.03	0.00–0.08	Small

^a, b,c^ indicates statistically significant (*p* < 0.05) Bonferroni-adjusted differences between group pairs

Multiparous women were observed to have significantly higher total Ger-BSS-R scores, and SE sub-scale scores compared to primiparous women. No other statistically significant differences were observed as a function of parity (Table 7).

**Table 7:** Comparison of Ger-BSS-R total and sub-scale scores differentiated by parity. Standard deviations are in parentheses, degrees of freedom = 216

BSS-*R* Scale	Primiparous (*N* = 112)	Multiparous (*N* = 106)	95% CI	t	*p*	Hedges g	Hedges g (95%CI)	Effect size
Stress	7.14 (3.42)	8.69 (3.38)	0.64–2.46	3.35	< 0.001	0.45	0.18–0.72	Small
Attributes	3.40 (2.20)	3.95 (2.21)	-0.04–1.14	1.85	0.07	0.25	-0.02–0.52	Small
Quality	14.32 (1.87)	14.08 (1.94)	-0.75–0.27	0.92	0.36	0.12	-0.38–0.44	Negligible
Total score	24.87 (5.50)	26.73 (6.21)	0.03–3.43	2.34	0.02	0.32	0.05–0.58	Small

## Discussion

The findings from the current translation and validation study present a complex picture in terms of interpretation and application. Firstly, the Ger-BSS-R was found to offer a good fit to the tri-dimensional measurement model of the founder BSS-R [17]. Further, the correlational characteristics between sub-scales and the total score are similar to those observed in the original English-language version. Thus far, from a measurement model perspective, the Ger-BSS-R can be considered equivalent to the original UK version. Additionally, the Ger-BSS-R was found to have excellent divergent and convergent validity characteristics.

The KGDV evaluation was also found to be broadly consistent with other translation and validation studies of the BSS-R, with multiparity being associated with higher BSS-R scores, which indicates higher birth satisfaction. In addition, an NVD is associated with comparatively higher BSS-R scores compared to deliveries with required interventions. However, an unusual finding was the observation that QC sub-scale scores were significantly higher in the NVD group compared to elective CS. One caveat to the statistically significant differences observed between groups is that the effect sizes were small. Therefore, it should be noted that in spite of these significant differences, the translation of these observations in terms of practical clinical findings, for example, practice change recommendations, would be premature at this juncture. Previous translation and validation studies have observed NVD and elective CS BSS-R sub-scale scores to be similar. Therefore, the current observations are of interest. One factor that may have potential impact in this regard, is defining characteristics of the population, and namely high-risk pregnancy. Almost invariably, BSS-R translation and validation studies to date have utilized low-risk populations, therefore, less is known about the characteristics of the BSS-R in high-risk groups. Thus, replication of the current observation regarding the impact of delivery type is necessary in other populations with high-risk pregnancy to confirm the veracity of these observations.

Since little work has been undertaken on the perception of birth experience in high-risk pregnancy, the impact of such on birth experience is particularly important. There are many potential reasons why high-risk pregnancy might impact birth experience and the mechanisms are complex and multifactorial. Fear of loss, elevated anxiety regarding deleterious maternal and infant outcomes, interactions between co-morbidities and the woman’s psychological set, including stress, anxiety, depression and self-efficacy can contribute to an altered birth experience. For example, though a woman may be aware of an increased risk, which consequently may be a source of anxiety, a specifically tailored plan of care may mediate such anxieties and contribute to a more positive birth experience. In contrast, a woman without a pre-existing medical condition but experiencing an unanticipated medical complication within the context of a standard care package may experience greater concern and a consequential impoverished birth experience. Thus, further research is required to understand the potential underlying mechanisms in the context of high-risk pregnancy and how these interact to impact both positively and deleteriously on birth satisfaction.

The internal consistency findings were observed to be lower than those observed in both the original BSS-R development study and several translations. This has implications in terms of the use of the sub-scales, since these are all below traditional threshold values for Cronbach’s alpha. The two-item WA sub-scale internal validity was acceptable according to the criteria of Clark and Watson (1995) [42]. However, the overall alpha for the total Ger-BSS-R scale was acceptable (> 0.70) and the issue in terms of the sub-scales may be population-specific rather than translation-specific. One possible interpretation is that women with pre-existing medical conditions face higher levels of stress during childbirth, as they are already burdened with disease-associated pain, disabilities, limitations or fears. This fact may also be causal for the lower Cronbach’s alpha in the sub-scale QC but is still not satisfactorily explained. Indeed, there are a number of potential possibilities that include, translation issues, population-specific issues, unaccounted for clinical variables or service delivery characteristics. This may not be a unitary cause and may represent an interaction between a number of possible influences. Further research is required to determine the potential factors involved. However, the comparatively lower score in the total Ger-BSS-R in the current study is an interesting finding, which requires further work-up to improve birth experiences in women with pre-existing conditions.

The notion that some items of the BSS-R may perform differently in the German context is difficult to address because the study is also contextualized within a high-risk pregnancy sample. A potential approach that could disentangle these issues and determine whether there are either language-based or population-based issues in specific item-responding is invariance analysis. Thus, a future study using this version of the BSS-R in a non-high-risk group would allow comparison with the current study data at an item-level within the measurement model of the BSS-R by investigation of invariance characteristics. If the BSS-R was found to be invariant between high risk and low risk groups then the findings from the current study could be deemed to generalize to the broader German population of women.

Given the current findings a question that potentially emerges is ‘should birth satisfaction be conceptualized differently in high-risk pregnancies’? Suggestions against this would be the good fit of the three-factor measurement model of the BSS-R to the current dataset. If birth satisfaction was fundamentally different in the context of high-risk pregnancy, then it would be anticipated that the measurement model would be a poor fit in these circumstances. Further, the theoretical model of the BSS-R as a measure of birth experience is based on the concept of a continuum across the range of potential birth experience, embracing all birthing circumstances. Thus, based on both the theoretical model and the current data, birth experience conceptualized within a tri-dimensional measurement model as assessed by the BSS-R would appear to be appropriate in this group. However, pregnancy and birth-specific circumstances may be anticipated to have an impact on birth experience and this be likely to impact on birth experience scores, and potentially differentially on specific sub-scales, thus mental health [47] and risk perception [48], may impact the SE sub-scale more than, for example, the QC sub-scale.

Furthermore, it was noted that the full range of responses was not utilized in several items, which can impact on internal consistency observations, particularly within sub-scales. Given the high-risk nature of pregnancies defining the group and the bespoke obstetric care provided, this may have had an impact on the participant response set to specific items.

Further, given that this particular translation of the BSS-R was developed in relation to use within the ICHOM guideline, which specifies the use of the BSS-R total score, the contextual use of the Ger-BSS-R as a single score would be supported by the total scale alpha being above criterion threshold.

Effective and adequate intrapartum communication with health care professionals is known to increase birth satisfaction [7]. To achieve respectful intrapartum care for every childbearing woman [49], the WHO has established an intrapartum care model for a woman-centered care [50]. With the translation and validation of the Ger-BSS-R, maternity care providers within the German-speaking population can know benefit from the use of the BSS-R to assess birth satisfaction and aim at changing health care provider-associated factors. As it is known that birth satisfaction can have a significant impact on maternal and neonatal health [1], efforts to improve women’s satisfaction with childbirth are urgently required. Through gathering birth satisfaction data, key areas for improvement can be identified within intrapartum settings. It is fundamental to acknowledge that the BSS-R is recommended as the key global clinical measure of birth satisfaction by the ICHOM Standard Set for Pregnancy And Childbirth [18].

This study is limited by the cohort’s characteristics, as birth experience may be influenced by women’s pre-existing medical conditions and consequently their high-risk pregnancies and childbirths. Furthermore, the Ger-BSS-R was applied in the framework of a questionnaire which also contained self-designed questions regarding the influence of the pre-existing condition on childbirth and motherhood. Furthermore, the timing of data collection may have influenced birth satisfaction scales, as women within 72 h postpartum are recovering from physical and emotional stress during childbirth. Other limitations are the high percentage of CS, which may be attributed to the specific cohort, and single-center study design.

## Conclusion

Our results have shown that the validated Ger-BSS-R is a robust instrument for health care professionals to assess childbearing women’s birth satisfaction. The purpose of developing country- and language-specific versions of the BSS-R is to create versions of the BSS-R for use within contextualized populations to assess and improve quality of care provided. This study is the first to assess birth satisfaction via the BSS-R in women with pre-existing medical conditions facing high-risk pregnancies and childbirths. Although the primary purpose of this study was the validation of the German version of the BSS-R, findings show interesting differences in birth satisfaction in this extraordinary cohort. While women in this study seem to face higher levels of stress and lower satisfaction with quality of care, the possible causality with the presence of a pre-existing medical condition needs to be further investigated.

## Electronic supplementary material

Below is the link to the electronic supplementary material.

Supplementary Material 1

## Data Availability

The BSS-R is openly available to researchers and evaluators of care delivery. Hollins Martin and Martin continue to work with teams at an international level to translate and validate population specific versions of the BSS-R, which allows clinical teams world-wide to produce a context specific robust tool for detailed projects. The newly validated version of the Ger-BSS-R will be made available free of charge at https://www.bss-r.co.uk. For permission and copy request, please contact Professor Caroline J. Hollins Martin at c.hollinsmartin@napier.ac.uk.

## Abbreviations

ANOVA: Analysis of variance
BSS-R: Birth Satisfaction Scale-Revised
CFA: Confirmatory Factor Analysis
CFI: Comparative Fit Index
CS: Cesarean section
ForMaT: ‘Forschung Maternale Medizin’-Trial name
Ger-BSS-R: German Version of the BSS-R
ICHOM: International Consortium for Health Outcomes Measurement
KGDV: Known-Groups Discriminant Validity
PRO: Patient-reported outcome
QC: BSS-R sub-scale for assessing ‘quality of care provision’
RMSEA: Root mean squared error or approximation
SD: Standard deviation
SE: BSS-R sub-scale for assessing ‘stress experienced during labor’
SRMR: Square root mean residual
NVD: Normal vaginal delivery
WA: BSS-R sub-scale for assessing ‘women’s personal attributes’
WHO: World Health Organization
