# Supplementary material for: Measurement properties of the German version of the birth satisfaction scale-revised (BSS-R) in women with pre-existing medical conditions and high-risk pregnancy
Source: BMC Pregnancy Childbirth. 2025 Apr 24;25:488. doi: 10.1186/s12884-025-07571-7 (PMC12023544; doi:10.1186/s12884-025-07571-7)
Supplement: Supplementary file 1 — Supplementary Material 1 [file 12884_2025_7571_MOESM1_ESM.docx]

Supplementary Material

Table S 1: Overview of the available translations and validations of the BSS-R.

| Language | Country | Author | Year | Reference |
| --- | --- | --- | --- | --- |
| Arabic | Saudi Arabia | D’Sa et al. | 2024 | (1) |
| Australian English | Australia | Jefford et al. | 2023 | (2) |
| American English | U.S. | Burduli et al. | 2017 | (3) |
| Brazilian Portuguese | Brazil | Ferrari et al. | 2021 | (4) |
| Czech | Czech Republic | Ratislavová et al. | 2024 | (5) |
| Dutch | Netherlands | Emmens et al. | 2021 | (6) |
| Greek | Greece | Vardavaki et al. | 2015 | (7) |
| Hebrew | Israel | Skvirsky et al. | 2020 | (8) |
| Hindi | India | Tiwari et al. | 2023 | (9) |
| Hungarian | Hungary | Abrán et al. | 2024 | (10) |
| Igbo | Nigeria | Anikwe et al. | 2022 | (11) |
| Iranian | Iran | Nasiri et al. | 2020 | (12) |
| Italian | Italy | Nespoli et al. | 2021 | (13) |
| Japanese | Japan | Tezuka et al. | 2023 | (14) |
| Polish | Poland | Pawlicka et al. | 2024 | (15) |
| Portuguese | Portugal | Moreira et al. | 2023 | (16) |
| Slovakian | Slovakia | Škodová et al. | 2019 | (17) |
| Spanish | Spain | Romero-Gonzalez et al. | 2019 | (18) |
| Swedish | Sweden | Grundström et al. | 2023 | (19) |
| Turkish | Turkey | Göncü Serhatlıoğlu et al. | 2018 | (20) |
| Urdu | Pakistan | Zafar et al. | 2021 | (21) |
| Vietnamese | Vietnam | MacVicar et al. | 2024 | (22) |

References:

1. D’Sa JL, Jahlan IO, Alsatari ES, Zamzam S, Martin CR. Psychometric properties of a Saudi Arabian version of the Birth Satisfaction Scale-Revised (BSS-R). Belitung Nurs J. 2024 Feb 28;10(1):105–13.

2. Jefford E, Hollins Martin CJ, Martin CR. Development and validation of the Australian version of the Birth Satisfaction Scale-Revised (BSS-R). Journal of Reproductive and Infant Psychology. 2018 Jan;36(1):42–58.

3. Burduli E, Barbosa-Leiker C, Fleming S, Hollins Martin CJ, Martin CR. Cross-cultural invariance of the Birth Satisfaction Scale-Revised (BSS-R): comparing UK and US samples. Journal of Reproductive and Infant Psychology. 2017 May 27;35(3):248–60.

4. Ferrari RB, Martin C, Hollins Martin C, De Souza FG, Clini JV, Onofre LBO, et al. Translation of the UK-Birth-Satisfaction-Scale-Revised (BSS-R) into Brazilian (Portuguese) and description of initial measurement properties. The Journal of Maternal-Fetal & Neonatal Medicine. 2024 Mar 6;35(25):6373–9.

5. Ratislavová K, Hendrych Lorenzová E, Hollins Martin CJ, Martin CR. Translation and validation of the Czech Republic version of the Birth Satisfaction Scale-Revised (BSS-R). Journal of Reproductive and Infant Psychology. 2024 Jan;42(1):78–94.

6. Emmens B, Hollins Martin CJ, Martin CR. Translation and validation of the Dutch version of the Birth Satisfaction Scale-Revised (BSS-R). Journal of Reproductive and Infant Psychology. 2023 Mar 15;41(2):213–27.

7. Vardavaki Z, Hollins Martin CJ, Martin CR. Construct and content validity of the Greek version of the Birth Satisfaction Scale (G-BSS). Journal of Reproductive and Infant Psychology. 2015 Oct 20;33(5):488–503.

8. Skvirsky V, Taubman–Ben-Ari O, Hollins Martin CJ, Martin CR. Validation of the Hebrew Birth Satisfaction Scale – Revised (BSS-R) and its relationship to perceived traumatic labour. Journal of Reproductive and Infant Psychology. 2020 Mar 14;38(2):214–20.

9. Tiwari SK, Murry L, Joshi P, Tallanao T, Zined R, Hollins Martin CJ, et al. Translation and validation of the Hindi‐Indian version of the Birth Satisfaction Scale‐Revised. J of Obstet and Gynaecol. 2023 Mar;49(3):938–45.

10. Abrán H, Kovács K, Horvát Z, Erőss E, Hollins Martin CJ, Martin CR. Translation and validation of the Hungarian version of the Birth Satisfaction Scale-Revised (BSS-R). Midwifery. 2024 May;132:103983.

11. Anikwe C, Osita US, Mbanefo PO, Asiegbu OG, Nnadozie UU, Eleje GU, et al. The Birth Satisfaction Scale: Igbo adaptation, validation, and reliability study [Internet]. 2022 [cited 2024 May 13]. Available from: https://www.qeios.com/read/GOVO55

12. Nasiri S, Kariman N, Ozgoli G. Psychometric properties of the Iranian version of Birth Satisfaction Scale-Revised. J Res Med Sci. 2020;25(1):90.

13. Nespoli A, Colciago E, Fumagalli S, Locatelli A, Hollins Martin CJ, Martin CR. Validation and factor structure of the Italian version of the Birth Satisfaction Scale-Revised (BSS-R). Journal of Reproductive and Infant Psychology. 2021 Oct 20;39(5):516–31.

14. Tezuka A, Hiroyama N, Suzuki M, Matsuoka M, Martin CJH, Martin CR. Translation and validation of the Japanese version of the Birth Satisfaction Scale‐Revised. Japan Journal Nursing Sci. 2024 Jan;21(1):e12569.

15. Pawlicka P, Wróbel W, Baranowska B, Macewicz D, Olech M, Hollins Martin CJ, et al. Translation and validation of the Polish-language version of the Birth Satisfaction Scale-Revised (BSS-R) and its relationship to the type of delivery and the baby’s Apgar score. Health Psychology Report [Internet]. 2024 Jun 19 [cited 2024 Dec 16]; Available from: https://hpr.termedia.pl/Translation-and-validation-of-the-Polish-language-version-of-the-Birth-Satisfaction,186231,0,2.html

16. Moreira H, Hollins Martin CJ, Martin C. Factor structure and psychometric properties of the Birth Satisfaction Scale–Revised in Portuguese postpartum women. Journal of Reproductive and Infant Psychology. 2023 Feb 14;1–16.

17. Škodová Z, Nepelová Z, Grendár M, Bašková M. Psychometric properties of the Slovak version of the Birth Satisfaction Scale (BSS) and Birth Satisfaction Scale-Revised (BSS-R). Midwifery. 2019 Dec;79:102550.

18. Romero-Gonzalez B, Peralta-Ramirez MI, Caparros-Gonzalez RA, Cambil-Ledesma A, Hollins Martin CJ, Martin CR. Spanish validation and factor structure of the Birth Satisfaction Scale-Revised (BSS-R). Midwifery. 2019 Mar;70:31–7.

19. Grundström H, Martin CJH, Malmquist A, Nieminen K, Martin CR. Translation and validation of the Swedish version of the Birth Satisfaction Scale-Revised (BSS-R). Midwifery. 2023 Sep;124:103745.

20. Göncü Serhatlıoğlu S, Karahan N, Hollins Martin CJ, Martin CR. Construct and content validity of the Turkish Birth Satisfaction Scale – Revised (T-BSS-R). Journal of Reproductive and Infant Psychology. 2018 May 27;36(3):235–45.

21. Zafar S, Tayyab F, Liaqat A, Sikander S, Hollins Martin CJ, Martin CR. Translation and Validation of the Birth Satisfaction Scale-Revised in Urdu for Use in Pakistan. Int J Childbirth. 2021 Jun 1;11(2):72–83.

22. MacVicar S, Jordan A, Vu H, Tran HN, Greig Y, Thi Tuyet Nguyen H, et al. Translation and validation of Vietnamese version of the Birth Satisfaction Scale-Revised (BSS-R). Journal of Reproductive and Infant Psychology. 2024 Apr 9;1–14.
